# Rituximab in the treatment of anti-HMGCR immune-mediated necrotizing myopathy: Two cases successfully treated

**DOI:** 10.1515/rir-2025-0014

**Published:** 2025-07-01

**Authors:** Giulia Micheli, Lorenzo Salvati, Boaz Palterer, Emanuele Vivarelli, Alessio Mazzoni, Nila Volpi, Alessandra Vultaggio, Andrea Matucci, Lorenzo Cosmi, Daniele Cammelli, Paola Parronchi

**Affiliations:** Department of Experimental and Clinical Medicine, University of Florence, Florence, Italy; Flow Cytometry Diagnostic Center and Immunotherapy, Careggi University Hospital, Florence, Italy; Neurology and Neurophysiopathology Unit, Department of Medical, Surgical and Neurological Sciences, University of Siena, Siena, Italy; Immunoallergology Unit, Careggi University Hospital, Florence, Italy; Immunology and Cell therapy Unit, Careggi University Hospital, Florence, Italy

Dear Editor,

Anti-HMGCR-positive myositis is an immune-mediated necrotizing myopathy (IMNM) characterized by the presence of antibodies directed against hydroxymethylglutaryl-CoA reductase (HMGCR), a key enzyme involved in cholesterol metabolism. Clinically, it presents with symmetric proximal muscle weakness, markedly elevated serum creatine kinase (CK) levels, abnormal findings on electromyography (EMG) and histopathological evidence of muscle fibre necrosis with macrophage infiltrate. A history of statins exposure is often reported, although the disease can also develop in statin-naïve individuals.^[1,2]^ Myositis persists even after drug discontinuation and requires immunosuppressive/immunomodulatory therapies, mainly represented by glucocorticoids, methotrexate (MTX) and high-dose intravenous immunoglobulins (IVIG).^[3]^ Although the response to treatment is generally good, some patients do not respond to initial therapy or relapse after steroid discontinuation. Rituximab (RTX) has been successfully used as second-line therapy, due to the putative pathogenic role of anti-HMGCR antibodies.^[4,5]^ Herein, we report two patients with anti-HMGCR-positive IMNM successfully treated with rituximab, in addition to standard therapy.

A 57-year-old man (case 1) and a 70-year-old woman (case 2) presented with progressive asthenia and limb muscle weakness. Both showed significantly elevated serum CK levels (12,946 and 11,344 U/L, respectively; reference range 39–308 U/L). Only case 2 had a history of statin use, but both had taken red yeast rice supplements prior to symptom onset. EMG revealed myositic and myopathic changes in both patients. In case 1, magnetic resonance image (MRI) was showed hyperintensities on FAT-suppressed images (Figure 1A). Anti-HMGCR antibodies were strongly positive on an enzyme-linked immunosorbent assay (ELISA) in both cases (315 and 402 U, respectively; reference range < 20 U). Initial treatment included high-dose intravenous steroids and IVIG followed by oral high-dose corticosteroids and MTX, with a rapid CK reduction and progressive improvement of muscle strength. However, CK and anti-HMGCR antibody levels remained elevated, and symptoms persisted, especially in case 1. In this patient a muscle biopsy was performed and discrete inhomogeneity in fibres calibre, nuclear internalizations, isolated nicotinamide adenine dinucleotide (NADH)-positive hypotrophic and diverse regenerating fibres, diffuse modest CD68 expression and increased expression of membrane attack complex (MAC) and major histocompatibility complex (MHC) class I on cellular surfaces in a picture of myopathic damage were found. For this reason, RTX therapy was proposed (1000 mg two weeks apart and then every 6 months for 3 times). Following RTX therapy, case 1 achieved full recovery, with normalization of CK, complete negativization of anti-HMGCR antibodies, resolution of MRI findings, and discontinuation of IVIG (Figure 1B, 2A-B). No adverse events occurred during and after treatment. Case 2 improved clinically, discontinued MTX and IVIG, and achieved reduction of anti-HMGCR antibody levels (Figure 2C-D). In this patient, a mild IgG1 deficiency occurred after RTX, without increased infections.

**Figure 1:**
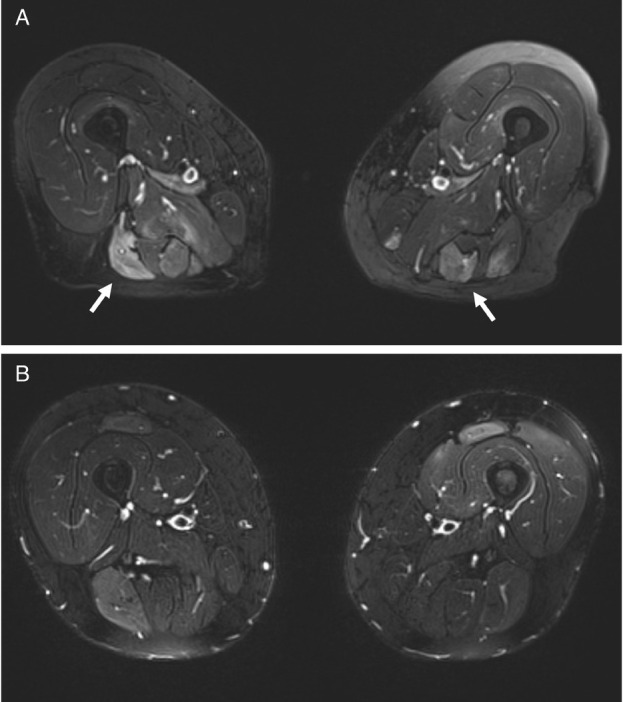
MRI scans of the thighs before and after rituximab therapy in Case 1. (A) Axial T2-weighted images of the upper thigh (December 2020) showing extensive areas of hyperintensity with muscle edema (white arrows). (B) Follow-up axial T2-weighted images (May 2022) of the same region demonstrate marked resolution of the previously observed edema.

**Figure 2:**
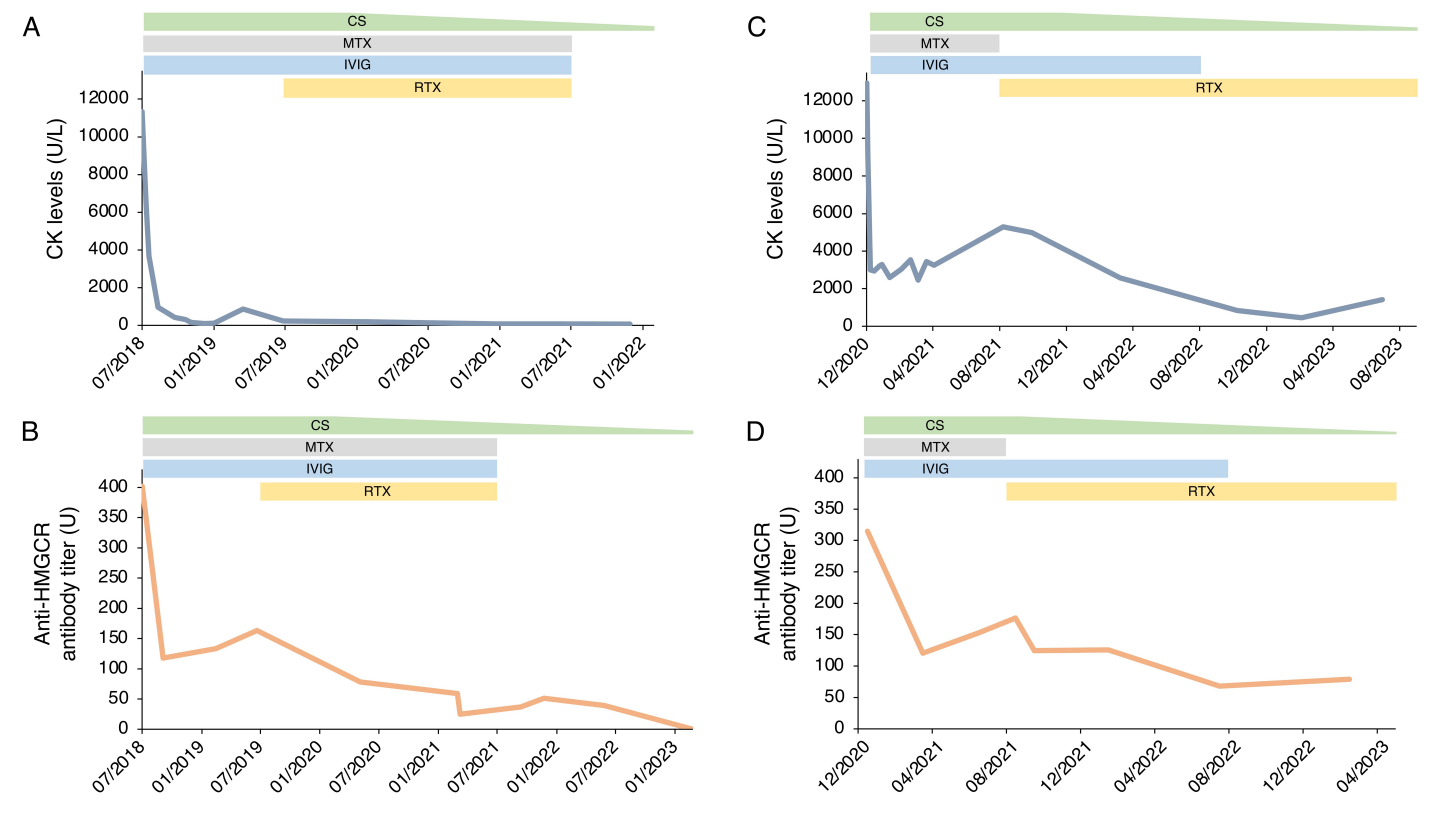
Disease progression in Case 1 and Case 2: longitudinal evaluation of CK levels (A-C) and anti-HMGCR antibody titers (B-D) in relation to treatment. CK levels (U/L) and anti-HMGCR antibody titers (U) are shown over time for Case 1 (panel A-B) and Case 2 (panel C-D). Reference ranges CK: 39-308 U/L; anti-HMGCR antibody titer: <20 U. CK, creatine kinase; CS, corticosteroid; MTX, methotrexate; IVIG, intravenous immunoglobulin; RTX, rituximab.

Anti-HMGCR myopathy is a rare subtype of IMNM.^[6,7]^ To date, prospective studies and randomized clinical trials specifically addressing the optimal therapeutic approach for this entity are limited. Anti-HMGCR antibody titres have been reported to correlate with disease severity, supporting a potential pathogenic role.^[8,9]^ Accordingly, B-cell depletion with RTX has emerged as a promising effective therapeutic strategy. However, published data on RTX efficacy in this subset of myopathies remain limited with currently no consensus regarding the optimal RTX protocol.^[10,11]^ In conclusion, the few retrospective studies published to date show substantial heterogeneity in the patient characteristics, treatment regimens, timing of RTX initiation, and biomarkers used to monitor response. Our findings contribute to the growing evidence supporting the use of RTX in anti-HMGCR-positive IMNM and emphasize the urgent need for prospective randomized clinical trials to evaluate its efficacy and safety in this context.

## References

[1] Mohassel P, Mammen AL (2018). Anti-HMGCR Myopathy. J Neuromuscul Dis.

[2] Giudizi MG, Cammelli D, Vivarelli E (2016). Anti-HMGCR antibody-associated necrotizing myopathy: diagnosis and treatment illustrated using a case report. Scand J Rheumatol.

[3] Allenbach Y, Mammen AL, Benveniste O (2018). 224th ENMC International Workshop:: Clinico-sero-pathological classification of immune-mediated necrotizing myopathies Zandvoort, The Netherlands, 14–16 October 2016. Neuromuscul Disord.

[4] Landon-Cardinal O, Allenbach Y, Soulages A (2019). Rituximab in the Treatment of Refractory Anti-HMGCR Immune-mediated Necrotizing Myopathy. J Rheumatol.

[5] Silva SP, Eugénio G, Pinto M (2024). Clinical and persistent remission in anti-HMGCR immune-mediated necrotizing myopathy to a single cycle of rituximab - a case-based review.” “Clinical and persistent remission in anti-HMGCR immune-mediated necrotizing myopathy to a single cycle of rituximab – a case-based review. ARP Rheumatol.

[6] Selva-O’Callaghan A, Alvarado-Cardenas M, Pinal-Fernández I (2018). Statin-induced myalgia and myositis: an update on pathogenesis and clinical recommendations. Expert Rev Clin Immunol.

[7] Khoo T, Chinoy H (2023). Anti-HMGCR immune-mediated necrotising myopathy: Addressing the remaining issues. Autoimmun Rev.

[8] Werner JL, Christopher-Stine L, Ghazarian SR (2012). Antibody levels correlate with creatine kinase levels and strength in anti-3-hydroxy-3-methylglutaryl-coenzyme A reductase-associated autoimmune myopathy. Arthritis Rheum.

[9] Martinez-Lopez D, Corrales Selaya C, Prieto-Peña D (2023). Anti-HMGCR Autoantibody Levels in the Follow-up of Statin-induced Immune-mediated Necrotizing Myopathy: Multicentric Study of 24 Patients [abstract]. Arthritis Rheumatol.

[10] Zhang W, Prince HM, Reardon K (2019). Statin-induced anti-HMGCR antibody-related immune-mediated necrotising myositis achieving complete remission with rituximab. BMJ Case Rep.

[11] Gupta S, Rakhra A, Thallapally V (2021). Rituximab use for refractory anti-HMGCR immune-mediated necrotizing myopathy: A case report. Intractable Rare Dis Res.

